# Localizing the United Nations Sustainable Development Goals to rural communities in America through university extension programmes

**DOI:** 10.1002/nop2.337

**Published:** 2019-07-23

**Authors:** Sarah Oerther

A nurse with a global perspective understands the benefits of local action and a global worldview. That is why nurses around the world are working to address the United Nations Sustainable Development Goals (SDGs). The SDGs consist of 17 goals and 169 targets, including ensuring healthy lives and gender equality (United Nations, 2015). In 2015, during the United Nations (UN) Sustainable Development Summit, the leaders of over 150 countries came together at the UN headquarters in New York to formally adopt the SDGs. Through a non‐binding collective agreement, these historic goals serve as a catalyst for action by national governments – with support from the international community – to achieve shared well‐being and prosperity by 2030 (United Nations, 2015).

At the global level, support for the SDGs includes new partnerships among governments and the private sector, academia, civil society and citizens. At the local level, adopting the SDGs is simple, but implementation is difficult because existing resources are already deployed in an attempt to accomplish previous development plans. Particularly, for resource‐limited rural communities in America, we will need to find ways to prioritize, customize and manage the implementation of the SDGs. Fortunately, the SDGs provide a blueprint for nurses and other community leaders to advance local economic, civic and environmental imperatives. Localizing the SDGs gives nurses and other community leaders in American rural communities an incentive to play a prominent role in solving the world's problems while solving their own.

As a nurse in my rural community in America, one of the ways I am addressing the SDGs is by taking a leadership role in a local university extension council. Formalized in 1914 through the Smith Lever Act, extension programmes are a partnership between the United States Department of Agriculture and land‐grant colleges and universities to bring the benefits of higher education to average citizens throughout the state (United States Department of Agriculture, 2019). One of the major activities of extension programmes is offering classes to local residents interested in learning new information or skills. Extension services provide training to non‐matriculated students – to farmers and other residents of rural communities, as well as to people living in urban areas. Through these classes, knowledge is transferred from higher education directly to local residents to create positive changes in the community (United States Department of Agriculture, 2019).

Extension programmes were created to provide training to communities. In contrast to accredited degree programmes, such as 2‐year associate, 4‐year baccalaureate and advanced degrees such as masters, doctorate or professional degrees, extension programmes are not intended to provide academic credit towards a degree. Individuals take classes offered by their local extension office in order to advance their knowledge in a subject. Since extension classes do not provide post‐secondary education credit of any kind, classes are afforded a higher degree of informality and flexibility as compared to classes offered to matriculated students (United States Department of Agriculture, 2019).

Extension programmes throughout America and the SDGs hold in common commitments to end poverty, achieve economic prosperity, and reduce inequality and injustice while promoting health and wellness. As a set of time bound, outcome‐based targets, the SDGs give meaning to metrics (United Nations, 2015). It is rare for local elected leaders and their communities to commit to the level of specificity contained in SDG targets. For example, while many community programmes, including extension services, might endorse a commitment to reducing poverty, it can be much more powerful – and motivating – to commit to reducing poverty by 50 per cent by 2030 (United Nations, 2015).

This type of data‐driven goal setting is embodied in the commitment made in the University of Missouri Extension strategic goals (University of Missouri System, 2018). For instance, the strategic goals for 2019–2029 include (a) improving the economy of Missouri by more than doubling economic impact – from $945 million–$2 billion dollars – over the next 10 years; (b) increasing the number of high school graduates participating in post‐secondary education – from the current 51%–60% – over the next decade; and (c) collaborating with partners to improve Missouri's national health ranking – from 40th−25th – over the next decade (University of Missouri System, 2018).

The first goal, which is to improve the economy of Missouri by more than doubling economic impact from $945 million–$2 billion dollars over the next 10 years, is aligned with SDG 8, which is to promote sustained, inclusive and sustainable economic growth; full and productive employment; and decent work for all (United Nations, 2015; University of Missouri System, 2018). As a nurse in a rural community and the chair of the programming committee for the Phelps County Extension Council, I am leading the way by overseeing the development of a programme to train local producers to safely handle produce and jams for local markets.

The second goal of increasing the number of high school graduates participating in post‐secondary education from the current 51%–60% over the next decade, aligns with SDG 4, which is to ensure inclusive and equitable quality education and promote lifelong learning opportunities for all (United Nations, 2015; University of Missouri System, 2018). As a nurse in a rural community and the chair of the programming committee for the Phelps County Extension Council, I am leading the way by supporting healthcare career fairs and events.

The third goal, collaborating with partners to improve Missouri's national health ranking from 40th–25th over the next decade, aligns with SDG 3, which is to ensure healthy lives and promote well‐being for all at all ages. In one of our small rural communities (Phelps county), there are an average of 54 deaths annually as a result of cancer (National Cancer Institute, 2015; United Nations, 2015; University of Missouri System, 2018). Opportunities to prevent deaths due to cancer include decreasing rates of smoking, increasing physical activity, increasing rates of human papillomavirus (HPV) and hepatitis B vaccinations, and increasing routine screening especially for breast, prostate and colon cancers. As a nurse in a rural community and the chair of the programming committee for the Phelps County Extension Council, I am leading the way by overseeing the development of a programme that provides peer support in establishing self‐care practices.

Worldwide advancements in health require action within local communities. Historically, the discipline of nursing has stressed practice, teaching and research. Based upon my personal experience as a local elected member of the Phelps County Missouri Extension Council, I call on nurses to undertake leadership roles in the creation and evaluation of healthcare programmes within their own communities in order to address the SDGs. To impact change, nurses should view community development opportunities as something they can influence, rather than something they ‘just’ support. International nurse leaders must be engaged in community development programme decisions and have a voice in implementation efforts. And future efforts should explore replication and adaptation of the local university extension councils as a global exemplar for equipping nurse leaders in community leadership so that nurses can address the SDGs within their own countries.

## References

[nop2337-bib-0001] National Cancer Institute . (2015). State cancer profiles. Retrieved from https://statecancerprofiles.cancer.gov/deathrates/index.php?stateFIPS=29&cancer=001&race=00&sex=0&age=157&type=death&sortVariableName=rate&sortOrder=default#results

[nop2337-bib-0002] United Nations . (2015). Sustainable Development Goals ‐ United Nations Sustainable Development. Retrieved from https://www.un.org/sustainabledevelopment/sustainable-development-goals/

[nop2337-bib-0003] United States Department of Agriculture . (2019). National Institute of Food and Agriculture. Retrieved from https://nifa.usda.gov/history

[nop2337-bib-0004] University of Missouri System . (2018). University for Missouri The Flagship of the Future. Retrieved from https://chancellor.missouri.edu/wp-content/uploads/2018/10/mu-strategic-plan-10042018.pdf

